# Somatic and *de novo* Germline Variants of *MEDs* in Human Neural Tube Defects

**DOI:** 10.3389/fcell.2021.641831

**Published:** 2021-03-04

**Authors:** Tian Tian, Xuanye Cao, Yongyan Chen, Lei Jin, Zhiwen Li, Xiao Han, Ying Lin, Bogdan J. Wlodarczyk, Richard H. Finnell, Zhengwei Yuan, Linlin Wang, Aiguo Ren, Yunping Lei

**Affiliations:** ^1^National Health Commission Key Laboratory of Reproductive Health, Institute of Reproductive and Child Health, Peking University, Beijing, China; ^2^Department of Epidemiology and Biostatistics, School of Public Health, Peking University, Beijing, China; ^3^Department of Molecular and Cellular Biology, Center for Precision Environmental Health, Baylor College of Medicine, Houston, TX, United States; ^4^Departments of Molecular and Human Genetics and Medicine, Baylor College of Medicine, Houston, TX, United States; ^5^Key Laboratory of Health Ministry for Congenital Malformation, Shengjing Hospital, China Medical University, Shenyang, China

**Keywords:** neural tube defects, MEDs, somatic variants, *de novo* variant, CRISPR/Cas9

## Abstract

**Background:**

Neural tube defects (NTDs) are among the most common and severe congenital defects in humans. Their genetic etiology is complex and remains poorly understood. The Mediator complex (MED) plays a vital role in neural tube development in animal models. However, no studies have yet examined the role of its human homolog in the etiology of NTDs.

**Methods:**

In this study, 48 pairs of neural lesion site and umbilical cord tissues from NTD and 21 case-parent trios were involved in screening for NTD-related somatic and germline *de novo* variants. A series of functional cell assays were performed. We generated a *Med12* p.Arg1784Cys knock-in mouse using CRISPR/Cas9 technology to validate the human findings.

**Results:**

One somatic variant, *MED12* p.Arg1782Cys, was identified in the lesion site tissue from an NTD fetus. This variant was absent in any other normal tissue from different germ layers of the same case. In 21 case-parent trios, one *de novo* stop-gain variant, *MED13L* p.Arg1760^∗^, was identified. Cellular functional studies showed that *MED12* p.Arg1782Cys decreased MED12 protein level and affected the regulation of *MED12* on the canonical-WNT signaling pathway. The *Med12* p.Arg1784Cys knock-in mouse exhibited exencephaly and spina bifida.

**Conclusion:**

These findings provide strong evidence that functional variants of *MED* genes are associated with the etiology of some NTDs. We demonstrated a potentially important role for somatic variants in the occurrence of NTDs. Our study is the first study in which an NTD-related variant identified in humans was validated in mice using CRISPR/Cas9 technology.

## Introduction

Neural tube defects (NTDs) are severe congenital defects caused by the failure of proper neural tube closure during early embryonic development. This group of malformations includes anencephaly, spina bifida, craniorachischisis, and encephalocele (Wallingford et al., 2013). The prevalence of these malformations places a heavy physical, mental, and economic burden on families and on society (Heazell et al., 2016; Zang et al., 2019).

Genetic and environmental factors are both involved in the etiology of NTDs. Genetic factors have long been known to play a vital role in the occurrence of NTDs (Copp et al., 2013). There are well over 300 naturally occurring, induced, or targeted mutations in murine genes that have been reported to be associated with NTD phenotypes. This no doubt contributes to the potential complexity of the underlying NTD genetics (Harris and Juriloff, 2007). However, many of these mouse NTD genes have not been critically evaluated as candidate genes for their association with human NTDs. Gene variants in fetuses may be inherited from parents or can occur *de novo*. These *de novo* variants may arise at a very early stage of embryonic development, affecting virtually all cells. It is also possible for these *de novo* variants to occur at a later developmental stage, affecting cells confined in a specific organ. These are referred to as somatic variants.

The somatic variants refer to a postzygotic change that can alter more than one group of cells with different genotypes in an organism, rather than a change in the DNA in a single fertilized egg. Somatic (non-germline) mutations are less likely to be passed on as well as not being inherited from either parent (Poduri et al., 2013). The role of somatic variants is not only well documented in cancer patients, but they have also been described in several neurodevelopmental disorders, such as McCune–Albright syndrome (Weinstein et al., 1991), the Sturge–Weber syndrome (Shirley et al., 2013), the Proteus syndrome (Lindhurst et al., 2011), and select brain malformations (Poduri et al., 2012; Riviere et al., 2012). These studies indicate that the nervous system is a highly susceptible region to somatic mutation (Muotri et al., 2005). However, there were only limited literature citations of studies reporting an association between a somatic variant with the development of an NTD. Using mouse models, Galea et al. (2018) reported that a somatic *Vangl2* deletion in neuroepithelial cells as well as surface ectodermal cells causes spina bifida in mice. In our recent publication, we described somatic variants in some key PCP genes (e.g., *VANGL1* and *FZD6*) in neural tissue that are associated with human NTDs, suggesting a potentially important role that somatic variants can play in the occurrence of human NTDs (Tian et al., 2020).

The Mediator complex (MED) is an essential molecular bridge that connects DNA-bound transcription factors to the RNA polymerase II machinery (Ding et al., 2008). The MEDs have significant regulatory effects on WNT signaling, which are known to have critically important signaling functions during neural tube closure (Rocha et al., 2010b). Med12 and Med13 are the two MED subunits in the regulatory domain. Various animal studies reveal that *Meds*, especially *Med12*, play a critical role in neural tube closure (Hong et al., 2005; Rau et al., 2006; Srivastava et al., 2019). Med12^hypo^ mouse embryos showed a down-regulation of Wnt5a and disturbed the normal localization of Prickle1, which are key PCP pathway genes, thus leading to severe developmental defects, including NTDs and heart defects (Rocha et al., 2010b). There have been no reports of any association between *MED* gene variants and human NTD risk.

Recent advances in genetic technologies has provided enormous opportunities to assess somatic mutations. In this study, we report on our efforts to identify both the germline and the somatic *MED* variants in human NTD cases. We initially involved 48 pairs of lesion site and umbilical tissues from NTD cases in screening the NTD-related somatic variants, and 21 NTD-case-parent trios, on which we performed sequencing assays to screen for *de novo* variants of MEDs. We subsequently performed cell functional assays to examine the effect of identified variants. Moreover, we generated a variant knock-in mouse model using CRISPR/Cas9 technology to validate the human findings, thus establishing substantial evidence for an association between altered *MED* genes as a risk factor for human NTDs.

## Materials and Methods

### Participants and Samples

The human samples were obtained from two independent cohorts.

The first cohort included 48 NTD cases that were recruited from five regions in the Shanxi province of northern China during the years 2011–2014. These fetuses were electively terminated following a prenatal diagnosis of an NTD. The tissues of the umbilical cord, skin, heart, kidney, thymus, liver, and pathological tissues of the spinal cord and brain of NTD cases were collected at pregnancy termination by experienced pathologists. The cohort included 11 fetuses with craniorachishisis, 15 fetuses with anencephaly, and 22 fetuses with spina bifida (Supplementary Table 1). We excluded cases with other congenital malformations. A detailed pathological examination of the fetuses was performed by experienced pathologists to confirm the diagnosis and the lesion site in each specimen. Remnant neural tissues and umbilical cord tissues were collected. Histopathology was performed on neural tissues by trained pathologists to confirm tissue identity (Supplementary Figure 1). All the available fetal and parental DNA were subjected to paternity testing to determine if all parental samples were from the actual biological parents.

The second cohort consisted of 21 NTD trios collected from Dell Children’s Medical Center at Central Texas (DCMC), TX, United States, 2011–2016. These NTD cases were all spina bifida cases. Saliva samples were collected using DNA Genotek Saliva DNA Collection Kit (OGR-250).

The first cohort was approved by the Institutional Review Board of Peking University, and written informed consent was obtained from the mothers before the invitation of all investigations. The second cohort was approved by the Institutional Review Board of the University of Texas at Austin. Written informed consent forms were obtained from the parents of all NTD cases.

### Ion Torrent Personal Genome Machine Sequencing

For the first cohort, the fetal DNA of tissues was extracted by using a QIAamp DNA Mini Kit Tissue kit (Qiagen, Germany). Maternal DNA from peripheral blood and paternal DNA from dried blood spots were extracted by using the QIAamp DNA Mini Kit Blood kit (Qiagen, Germany) and QIAamp DNA Mini Kit Bloodspot kit (Qiagen, Germany), respectively. The extracted DNA was electrophoresed to determine the degree of any degradation; then the genomic DNA was diluted to 5 ng/μL. The library for each sample was amplified in separate reactions. The purified DNA library was created by the use of Ion AmpliSeq^TM^ Library Kit 2.0. A water-in-oil response was performed on the Ion OneTouch^TM^ 200 Solutions Kit v2 Instrument (Life Technologies). Enrichment of Ion Sphere Particles (ISPs) was carried out on the Ion OneTouch ES instrument. The prepared samples were loaded onto a 318^TM^ sequencing chip (Life Technologies), and enriched ISPs were sequenced in the Ion PGM instrument.

### Whole Exome Sequencing

For the second cohort, all 21 NTD trios samples collected at DCMC were used for whole-exome sequencing (WES). WES was performed using the SureSelect Human All Exome 50 Mb Kit (Agilent Technologies, United States) with 150-bp paired-end read sequences generated on a HiSeq4000.

### Sanger Sequencing Validation

Sanger sequencing was performed to validate the non-synonymous variants in the target genes. NCBI/Primer-BLAST online tool was used to design the PCR primers (MED12_F: 5′-TTTTTGAGGGGTTGAAGCCG-3′; MED12_R:3′-CCCACA AACCAGAAACCGGA-5′; MED13L_F: 5′-GGTGTGAGCCAA CACATCCA-3′; MED13L_R: 3′-CCGTTTCCTTTTTCAGCTT GC-5′). Sequencing was conducted by the BigDye Terminator v3.1 Cycle Sequencing Kit and a 3130XL Genetic Analyzer (Applied Biosystems). The results were processed by Mutation Surveyor 4.0.8 software. Validated variants were examined to explore the source and distribution in samples of skin, heart, muscle, thymus, and lung from NTD cases or blood from parents of the cases when available. Parentage was confirmed using AmpFlSTR^®^ dentifiler^®^ Plus PCR Amplification Kit (Life Technologies, Foster City, CA, United States).

### Plasmids

pcDNA3.1 + /C-eGFP-*MED12* construct (clone ID: OHu19668) was purchased from Genscript Company (Piscataway, NJ 08854, United States). The mutated constructs of *MED12* p.Arg1782Cys were produced based on their wildtype plasmids using the GeneArt^®^ Site-Directed Mutagenesis System (Thermo Fisher Scientific, CAT#: A14604). All plasmids were validated by Sanger sequencing analysis. AP1, Top-flash, and pTKRenilla plasmids were obtained from Addgene (Cambridge, MA).

### Immunofluorescence Staining

Immunofluorescence staining was used to characterize the subcellular location of wildtype and mutated MED12. MDCK II cells were plated at a density of 3 × 10^5^ cells per ml onto 18 mm coverslips (Corning, Corning, NY) 20 h before transfection. The constructs of pcDNA3.1 + /C-eGFP-*MED12* (WT or mutant) were transfected into cells using Lipofectamine2000 (Invitrogen^TM^, Waltham, MA) according to the manufacturer’s protocol, separately. After 48 h culturing, cells were fixed and then stained with anti-rabbit-GFP (1:500, Abcam, MA, United States) antibody overnight. The nuclei of cells were stained with DAPI (300 ng/ml) for 2 min. Images were obtained with a Deconvolution microscope (Nikon T2).

### Western Blot Analysis

HEK293T cells were transfected with pcDNA3.1 + /C-eGFP-*MED12* (WT or mutant) using Lipofectamine 2,000 (Invitrogen, Waltham, MA). After 48 h of culture, the cells were lysed with 1x NP40 Lysis buffer (Invitrogen^TM^) with cOmplete^TM^ ULTRA Tablets (Millipore Sigma) for 20 min. The protein lysates were immunoblotted with anti-GFP and anti-GAPDH overnight. IRDye^®^ 800CW goat anti-rabbit IgG secondary antibodies (LI-COR, Cambridge, UK) were subsequently used to stain the cells for 1 h. The images were captured by Odyssey^®^ (LI-COR).

### Luciferase Reporter Assays

To examine the effect of mutants on the WNT signaling pathway, we performed a luciferase assay. The TOPFlash plasmid was used to serve as a canonical Wnt pathway signaling marker (Veeman et al., 2003), while a pAP1-Luc plasmid was used to measure activation of the non-canonical Wnt/PCP pathway signaling (Lei et al., 2015). HEK293T cells were transfected with pcDNA3.1 + /C-eGFP-*MED12* (WT or mutant) along with TOPFlash or pAP1-Luc and Renilla-TK plasmid. Cells were lysed by passive lysis buffer (Promega, Madison, WI) 24 h after transfection. The luciferase activity was measured by the Dual-Luciferase Assay Kit (Promega, Madison, WI). The Biotek-2 plate reader was used to read the luminescence activity.

### RT-qPCR Assay

The RT-qPCR assay was performed to examine the transfection effect (Supplementary Figure 2). The MDCK-II cells were transfected with equivalent amounts of Ampicillin resistant GFP-MED12 plasmids. We examined the expression of GFP and Ampicillin. The GAPDH and β-actin were used as the internal reference. The relative expression of GFP and Ampicillin to GAPDH and β-actin were calculated. The Student’s *t*-test was used to compare the relative expression of plasmid RNA.

### CRISPR/Cas9 Editing on Mouse

Six-week-old female C57BL/6 mice were used to provide the embryos on which to perform CRISPR/Cas9 assay. All procedures with animals were consistent with the Guidelines on the Use and Care of Laboratory Animals for Biomedical Research published by the National Institutes of Health (no. 85-23, revised 1996), and the experimental protocol was reviewed and approved by the animal care IACUC of Baylor College of Medicine. Med12 sgRNA was purchased from Synthego (sequence: gcUgcUccUccgagcacUg.). Single strand DNA oligos were ordered from IDT [sequence:a^∗^g^∗^ccccccaagactgacaaaccCggggctgctcctccgagTacCga Agag**T**gcaaaaagaagtctaccaagggcaaaaaacgcagccagccagccaccaag^∗^a^∗^a (107bp)]. Cas9 protein was purchased from Thermo Fisher Scientific (Cat# A36496). The 6-week-old mice were injected with 30 units of pregnant mare serum gonadotropin (PMSG, Sigma-Aldrich), followed by an injection of 30 units of human chorionic gonadotropin (hCG, Sigma-Aldrich) 48 h later, and immediately mated with males. Zygotes were obtained the next day and cultured in KSOM (Millipore) at 37°C, 5% CO_2_ for 2 h, and were prepared for microinjection. Zygotes were injected with a mixture of Cas9 protein (20 ng/μL) and Med12 sgRNA (10 ng/μL), and Med12 SSODN (10 ng/μL) to the targeting site. Microinjections were performed in fertilized eggs using a Nikon Microinjection system under standard conditions. The injected zygotes were then transferred to pseudopregnant mice (32 zygotes per mouse for a total of 6 mice) to be carried to parturition. Mice were dissected at embryonic day 13 to screen the embryos for the presence of any malformations. NTDs and embryolethality were recorded.

The embryos are microinjected with the aid of an inverted microscope equipped with differential contrast optics, usually Nomarski or Hoffman objective lenses, and micromanipulators. The embryos are injected with the aid of two needles. The Holding needle anchors the embryo in place with suction supplied by a micrometer syringe. The Injection needle, which has a continuous flow of DNA being forced through its opening, allows the DNA to be introduced into the pronucleus. The DNA flow is given by positive pressure driven by a micrometer syringe and introduces 2 pl of DNA at a 2 ng/μl concentration. The injections were performed twice.

The Sanger DNA sequencing method was performed to confirm the genetic variants in the mice, while whole-exome sequencing was used to make sure that the NTD phenotypes of knock-in mice were caused by Med12 variants rather than other NTD-causing genes (Supplementary Figure 3).

### Bioinformatics and Data Analysis

In the first cohort, sequencing raw data from the PGM runs were analyzed by the Coverage Analysis and Variant Caller plugins, which are available within the Torrent Suite^TM^ Software from the PGM platform. Sequenced reads were visualized with the IGV tool using the hg19 human reference genome. The coverage information, identification of low-frequency variants, and variant annotation were achieved with the aid of the Ampliseq “Tumor-Normal” sample workflows within the Ion Reporter suite v4.6. Mutations with a depth ≥ of 100x, Minor Allele Frequency (MAF) in the control population < 1%, and *p*-value of ≤ 5 × 10^–5^ were included for further analysis. We designated umbilical cord tissue as a control for defining somatic mutations, as it is one of the earliest developed fetal tissues during embryonic development (Krzyzanowski et al., 2019). A mutation at lesion site tissue but not in the same individual’s umbilical cord tissue was defined as a somatic mutation (Knudson, 1971).

In the second cohort, the DNA sequences were aligned to hg19, and variants were identified through the GATK pipeline^1^. *De novo* variants were analyzed by using TrioDenovo (Wei et al., 2015). Variations were annotated with Anavar. All variants were prioritized by allele frequency, conservation, and predicted effect on protein function.

dbSNP^2^, GenomAD^3^ and ExAC^4^ databases were used to annotate the identified mutations. SIFT^5^ and PolyPhen2^6^ were used to predict whether the mutations were deleterious or not. Clustal-Omega 1.2.1 software^7^ was used to estimate the conservation of proteins between different species. Localization of the mutations in their protein domains was assessed by Pfam 32.0^8^. Mutation Surveyor 4.0.8 software and Minor Variant Finder Software were used to analyze Sanger sequencing data. Sequencing and the analysis of sequencing data were performed by each group of the authors independently. Due to ethical reasons, the raw sequencing data of one group was only accessed by this group’s authorized members.

SPSS20.0 software was used for analyzing the data of cell experiments. The relative protein level (mean ± SE) was analyzed with Student’s *t*-test. At the same time, the incidence of abnormally located cells between the WT and mutation groups was compared by using a Chi-square test. *P*-values < 0.05 were considered statistically significant.

## Results

### Sequencing Study on Human NTD Cohorts

**FIGURE 1:**
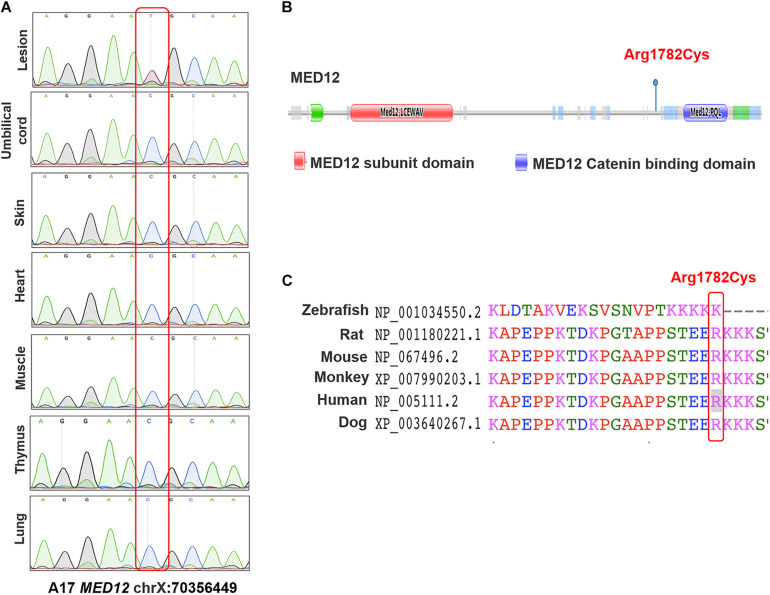
The identified variants of *MED12* in human samples. **(A)** Sanger sequencing map of the *MED12* c.5344C > T in different organs. **(B)** Conserved domains and the total scheme of the target genes and positions of the identified mutations. **(C)** A partial alignment of human target MED12 protein with other orthologous sequences.

The first cohort includes 48 pairs of neural lesion site and umbilical cord tissues from NTD affected fetuses. The cohort includes 45.8% male fetuses and 54.2% female fetuses. The mean gestational age was 24.5 weeks. We performed PGM sequencing to identify potential NTD-related somatic variants and validated the identified variants by using Sanger sequencing. As a result, we validated one heterozygous somatic variant of *MED12* c.5344C > T (p.Arg1782Cys) in the lesion site tissue of a terminated female fetus diagnosed with craniorachischisis (Figure 1A). This variant was absent from any other normal tissue from organs derived from different germ layers such as umbilical cord, skin, heart, muscle, thymus, and lung, indicating that this variant only occurred in neural tissue (Figure 1A). As Figure 1B depicts, *MED12* p.Arg1782Cys is not located in the known functional domains of MED12, but the amino acid change induced by the variant occurs at a highly conserved position in mammals (Figure 1C).

**FIGURE 2:**
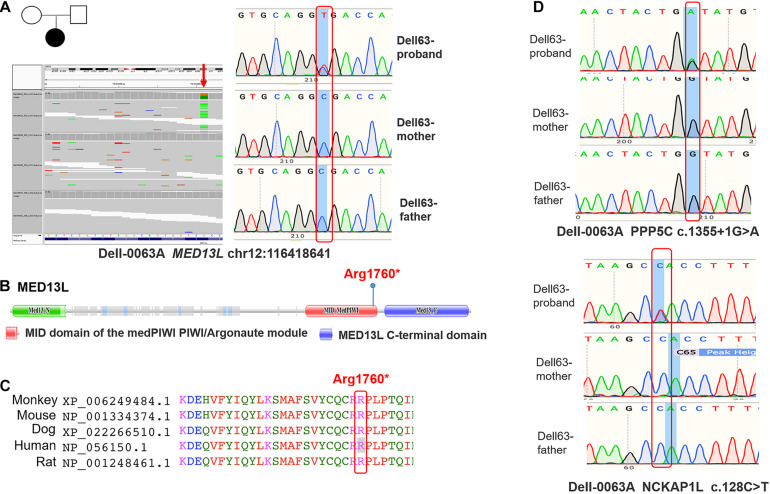
The identified variants of *MED13L* in human samples. **(A)** Whole-exome-sequencing map and Sanger sequencing map of *MED13L* c.5278C > T. **(B)** Conserved domains and the total scheme of the target genes and positions of the identified mutations. **(C)** A partial alignment of human target MED13L protein with other orthologous sequences. **(D)** The Sanger sequencing map of PPP5C and NCKAP1L genes.

In the second cohort consisting of 21 case-parent trios, we performed WES to identify the potential germline *de novo* variants of MEDs and performed Sanger sequencing assays to validate the identified variants. As **depicted in** Figure 2A, a heterozygous stop-gain variant of *MED13L* c.5278C > T (p.Arg1760^∗^) was detected in a female newborn diagnosed with spina bifida. This variant was absent in both parents, thus confirming its *de novo* nature. *MED13L* p.Arg1760^∗^ is located in the medPIWI domain of the MED13L protein, which is predicted to bind double-stranded nucleic acids, triggering the experimentally observed conformational switch in the CDK8 subcomplex that regulates the Mediator complex (Burroughs et al., 2013; Figure 2B). The mutant’s affected amino acid is highly evolutionarily conserved among different species (Figure 2C). Moreover, our patient with this variant also had a *de novo* deleterious variant of *NCKAP1L* c.128C > T (p.Pro43Leu) and a *de novo* splice variant of *PPP5C* c.1355 + 1G > A in its 11th exon (Figure 2D). Thus, we considered that possibly the latter two variants contributed in concert with the *MED* variant to produce the observed NTD phenotype.

### The Variant of MED12 p.Arg1782Cys Influences Cell Functions

**FIGURE 3:**
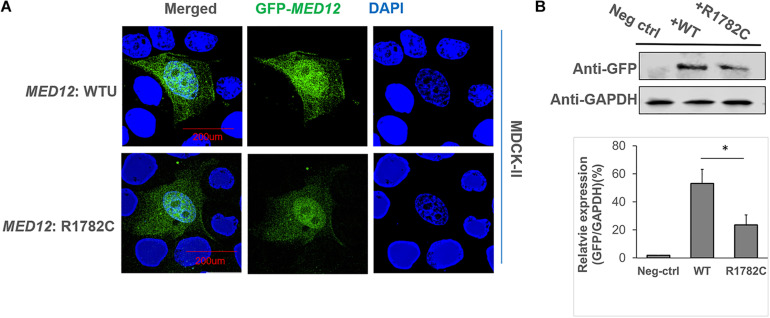
The influence of *MED12* p.Arg1782Cys on MED12 subcellular location and protein levels. **(A)** The subcellular localization of wildtype of MED12 and mutated MED12. MDCK-II cells were transfected with GFP-*MED12* (WT and mutants) for 48-h incubation and then were imaged under a deconvolution microscope. WTU: wildtype. The staining assay was performed twice. **(B)** The variant affected the protein level of MED12. HEK293T cells were transfected with GFP-*MED12* (WT and mutants) for 48 h, and then western blotting assays were performed to determine the protein levels. The predicted sizes of WT and mutant constructs are 260 KD. The western blotting assay was repeated three times, and *t*-tests on the relative protein levels to GAPDH were conducted to assess potential differences between WT and the mutant group. *p < 0.05. Ns: not significant. Three replicates of the transfections and western blotting assays were performed.

We initially examined the effect of the MED12 p.Arg1782Cys variant on MED12 subcellular localization. The constructs of *MED12* (WT and mutant) were overexpressed in MDCKII cell lines. As Figure 3A indicates, both the wildtype and MED12 mutant protein were expressed within the cell nucleus, indicating that the variants did not affect the subcellular location of MED12. However, the fluorescence of GFP signaling of mutated MED12 was reduced compared to the wildtype MED12 protein, implying the variant may affect the MED12 protein level. We subsequently performed a western blotting assay to examine the protein level. The western blotting assay was performed three times, and data analysis was carried out (Figure 3B). As a result, the protein coded for by the MED12 variant p.Arg1782Cys was less abundant than were the wildtype MED12 (*p* < 0.05), indicating that this mutation may decrease the MED12 expression or damage its protein stability.

**FIGURE 4:**
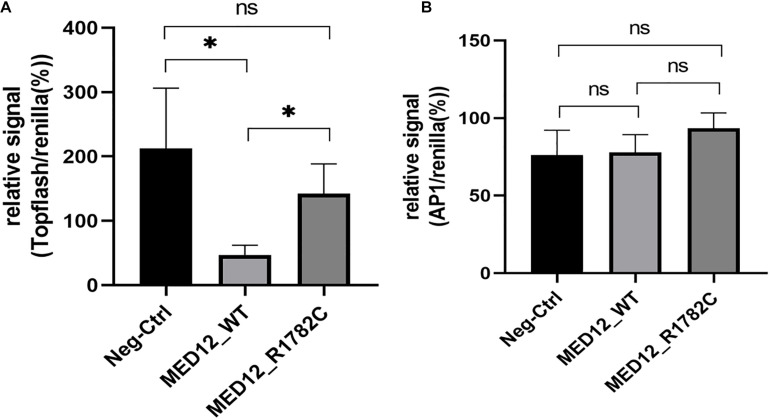
The influence of *MED12* p.Arg1782Cys on the expression of WNT signaling pathway. **(A)** The variant of *MED12* affected the non-canonical WNT signaling pathway. Luciferase assay was performed among HEK293T cells. Top-flash relative signal to Renilla represents the canonical WNT signaling. *p < 0.05. Ns: not significant. **(B)** The effect of the variant of *MED12* on the canonical WNT signaling pathway. Luciferase assay was performed among HEK293T cells. AP1 represents non-canonical WNT signaling.

A luciferase assay was used to investigate the influence of the variant on WNT signaling. The topflash reporter gene was used to measure canonical WNT signaling, while the pAP1-Luc reporter gene was used to represent non-canonical signals. The expression of canonical WNT signaling in the mutated *MED12* was significantly higher than in the *MED12* wildtype (*p* < 0.05). Considering that the variant of MED12 down-regulates the MED12 protein, we assume that the *MED12* p.Arg1782Cys might partially impair the regulatory effect of *MED12* on the canonical WNT signaling pathway by down-regulating the MED12 expression (Figure 4A). Although both the *MED12* wildtype and mutated variant up-regulated non-canonical WNT expression, there was no significant difference between the wildtype and mutated groups (Figure 4B).

### Med12 p.Arg1784Cys Knock-in Mice Present With NTD Phenotypes

CRISPR/Cas9 mutagenesis editing was employed to generate *Med12* p.Arg1784Cys (homologous position of p.Arg1782Cys in human) knock-in mice. *Med12*-targeting sgRNAs and CRISPR RNAs were microinjected into C57BL/6 N zygotes. The first time 124 embryos were injected and implanted into 4 recipient dams. No liveborn pups were observed. The second time, 200 embryos were collected from 16 C57/BL6 donors and injected. 192 injected embryos were implanted into 6 recipients (each recipient carried 32 embryos). Donor dams were killed and dissected at E12.5 (the injection day was designed as day E0.5). 12 viable embryos and 1 dead embryo were observed. 10 embryos were male, and 2 were female. For the 10 male embryos, 2 were MED p.Arg1784Cys knock-in hemizygotes with NTD phenotypes. The others were wildtype and no obvious structural malformations. The two female embryos were wildtype.

**FIGURE 5:**
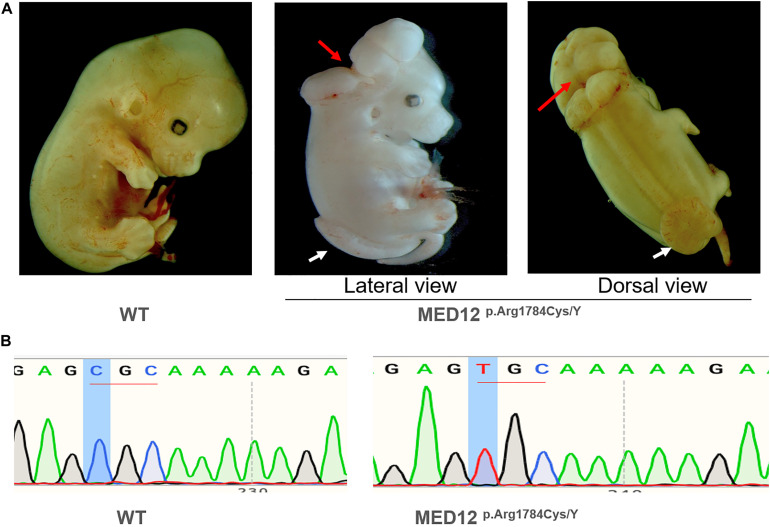
The knock-in of MED12 c.5344C > T (p.Arg1784Cys) on mouse embryogenesis. **(A)** The knock-in of *MED12* c.5344C > T (p.Arg1784Cys) produced mouse embryos displaying exencephaly and spina bifida phenotypes. The lesion site of exencephaly was indicated by the red arrow, while the spina bifida lesion site was indicated by the white arrow. The phenotypes of mice were observed and captured under a 6X microscope. **(B)** DNA Sanger sequencing on the CRISPR/Cas9 Mice.

As Figure 5A shows, the two hemizygous *Med12*^p.Arg1784Cys/Y^ mouse embryos exhibited NTD phenotypes, including exencephaly and spina bifida and curly tails. The result indicates that *Med12* p.Arg1784Cys identified in a human NTD patient can also cause NTDs in mice. The ensuing mice born from this injection were analyzed by DNA sequencing to ensure the integrity of the *Med12* p.Arg1784Cys knock-in (Figure 5B). Additionally, WES was carried out to make sure that *Med12* p.Arg1784Cys knock-in mice did not carry other previously reported NTD-related gene variants (Supplementary Figure 3).

## Discussion

Mediator (MED) is an evolutionarily conserved protein complex involved in the regulated transcription of nearly all RNA polymerase II-dependent genes (Sierecki, 2020). Mediator complex subunit 12 (*MED12*), located on the X chromosome (Philibert et al., 1999), and Mediator complex subunit 13-like (*MED13L)*, which is located on chromosome 12 (Musante et al., 2004), are two crucial subunits of the *MED* complex. MED12 is expressed throughout the central nervous system and in other tissues and is highly expressed during early embryonic development (Philibert et al., 1999), while MED13L is expressed in the embryonic brain, liver, and kidney, but not in the lung (Musante et al., 2004). These two proteins can combine with cyclin-dependent kinase 8 (CDK8) and Cyclin-C to form a MED12-MED13L-CDK8 complex. In this manner, it can regulate the Wnt/β-catenin and Notch signaling pathways so that they are either activated or repressed depending on the factors with which it interacts (Poot, 2020). A previous study reported that variants in *MED12* and *MED13L* were associated with neurodevelopmental disorders (Popp et al., 2017). *Med12* null mice show severe gastrulation defects, as stated in the Introduction, *Med12hypo* embryos have NTDs as do the mosaic females (Rocha et al., 2010b). In this study, we provided the first human evidence demonstrating that *MED12* and *MED13L* are also associated with an increased risk for human NTDs. We assume that variants of *MED12* and *MED13L* may disrupt the complex of MED12-MED13-CDK8, and this will impair the regular expression of WNT signaling pathway members, resulting in failed neural tube closure. Although the *de novo* stop-gain variant of *MED13L* p.Arg1760^∗^ was previously reported in an intellectual disability case (Smol et al., 2018), we also identified *de novo* variants of *PPP5C* and *NCKAP1L* in the same individual with the *MED13L* variant. PPP5C is a member of the protein phosphatase family (Xie et al., 2018), while *NCKAP1*L is the hematopoietic protein hem1 (Hromas et al., 1991). Although there has been no previously reported association of PPP5C or NCKAP1L with the development of NTDs, it was reported that PPP5C was a phosphatase of Dvl2, which has been implicated in neural tube closure (Xie et al., 2018). *NCKAP1*L is located at a rare folic acid-fragile site of DNA (Hromas et al., 1991), indicating these two genes play potentially important roles in the etiology of NTDs, and may have synergistic effects with MED13L that compromised neural tube closure. However, since our findings arose in a human DNA sequencing study, further validation in future studies is required.

Most of the previously published studies exploring human NTD-related gene variants have used blood and saliva samples and have focused on germline variants (Wolujewicz and Ross, 2019). Previous chimera studies of the loop-tail (*Vangl2*) mouse mutant that combined both mutant and wild type cells from all embryonic tissues of the embryo established that 16% of mutant cells determined the NTD phenotype within the sample (Ybot-Gonzalez et al., 2007). Those with high proportions of mutant cells develop NTDs, and those with low ratios develop normally (Musci and Mullen, 1990). An animal study showed that the mosaic expression of *Med12* in female mice is sufficient to produce progeny with congenital malformations such as exencephaly, spina bifida, craniorachischisis, split face, and curly tail phenotypes (Rocha et al., 2010a). One of our recent studies focusing on key PCP pathway genes, for which germline variants have been reported to have a role in human NTDs, found that somatic variants in specific PCP genes may also contribute to the NTD phenotype (Tian et al., 2020). These findings indicate the need to further investigate the role of somatic variants in other genes in the occurrence of NTDs. Therefore, in this study, we focused on genes reported to cause NTDs in mouse models but lacking evidence in humans. We investigated the somatic variants directly in neural tissues from NTD affected fetuses. As a result, one somatic variant of MED12 was identified and validated in the lesion site tissues of NTDs by PGM sequencing. Further functional studies revealed that this variant could impair MED12 protein levels, with adverse consequences for the WNT signaling pathway, indicating that the somatic variant p.Arg1782Cys of *MED12* may be causally associated with increased risk for human NTDs.

To explore the source and distribution of the identified somatic mutations, we utilized the DNA samples and performed sequencing in different tissues from the three primordial germ layers, including the epidermis, derived from the ectoderm; the heart and muscle tissues, which are mesodermal in origin; and the thymus and lungs, which are endodermally derived. The validated somatic variant of *MED12* p.Arg1782Cys was not present in samples from any of those normal tissues. It could only be recognized in neural tissues, with a 39.2% alternate-allele reads/counts frequency in PGM sequencing. As the human genome is diploid and the variant is supposed to be present in only one of two alleles, we infer that about 78% of cells carried the variant in the affected embryonic regions. This frequency is most likely high enough to disrupt normal neural tube closure. Furthermore, this mutation was identified in a case with craniorachischisis, and the tissues were obtained from the high spinal region where closure 1 was expected to occur, so we speculate that during development the MED12 mutation may only be present in neural tissue, not non-neural ectoderm.

Although hundreds of mouse models report NTD-related variants, it is rare for one of these variants to be validated in human NTD samples. On the other hand, human sequencing studies have identified many rare variants in NTD cases, but seldom have these genes been effectively recapitulated in an NTD mouse model. This study utilized a CRISPR/Cas9 mutagenesis editing approach and found that hemizygous variants of *Med12* p.Arg1784Cys caused NTD phenotypes in mice, which clearly support the results by Rocha and colleagues showing NTD in Med12 mosaic female mice (Rocha et al., 2010a). This is the first study that validates an NTD-related variant identified in humans by successfully recapitulating the NTD phenotype in mice by CRISPR/Cas9 technology, thus providing substantial evidence for the role of MED12 in the etiology of some human NTD cases.

There are some limitations in this study that need to be mentioned before any potential extrapolation of the findings. First, given that the spinal cord and brain tissues of the NTD affected fetuses are exposed to the amniotic fluid, it is conceivable that the variants could occur due to inflammatory (or teratogenic) effect on the neural tissue. Previous studies reported that neurodegeneration may cause several harmful outcomes, including DNA breaks (Suberbielle et al., 2013). Thus, it is possible that the variants described in this study may be the result of neural tissue degeneration. In practice, the neural tube tissue can only be obtained after the failed closure of the neural tube is confirmed, and at the time of pregnancy termination, therefore degeneration is inevitable. As a result, we cannot completely rule out the possibilities that the variants we identified were caused by tissue degeneration. However, several studies have reported that variants may affect cell proliferation and survival and contribute to neurodegeneration (McConnell et al., 2017; Leija-Salazar et al., 2018). Second, in PGM sequencing, we reported that approximately 78% cells carry the *MED12* p.Arg1782Cys variant. Considering that *MED12* is located on the X chromosome and female tissue is mosaic secondary to the random process of X-chromosome inactivation that may change the proportion of cells, even if 78% cells carry the variant, it doesn’t mean that all express the mutant form. Med12 mRNA expression data might resolve this point. However, as we mentioned above, we collected the samples of terminated fetuses from the hospitals; thus the collection of fresh tissue samples was extremely difficult. The RNA/protein often were severely degraded during the process of the termination, autopsy, and sample delivery. It is very challenging to investigate the expression form of the two mutants in the two patients. Moreover, in this study, we used a series of several cellular assays and a mouse model to show that the identified variant could impact protein function and cause murine NTDs. These results support our hypothesis that the mutations are causes, rather than consequences, of abnormal neural tube closure. Furthermore, we identified three *de novo* variants in a single human patient raising the possibility that interactions between MED13L, NCKAP1L, and PPP5C are contributing to the failure of the neural tube to close properly. This will be explored in future studies.

## Conclusion

Our findings represent the first time that functional variants of *MED* genes are associated with the occurrence of NTDs in humans. We also demonstrated a potentially important role of somatic variants in the occurrence of some NTDs. Further studies are required to explore the underlying interaction between *MED12* and *MED13L* in the development of NTDs and the somatic variant’s underlying mechanisms that interfere with normal neural tube closure.

## Data Availability Statement

Due to ethical and legal reasons, the datasets for this article are not publicly available. Requests to access the datasets for the first cohort should be directed to LW at linlinwang@bjmu.edu.cn, and the datasets for the second cohort and CRISPR/Cas9 should be directed to YL at yunping.lei@bcm.edu.

## Ethics Statement

The study protocol was approved by the Institutional Review Board of Peking University and by the Institutional Review Board of the University of Texas at Austin. Written informed consent was obtained from all of the mothers before the start of the investigation. Written informed consent was obtained from all of the mothers before the start of the investigation.

## Author Contributions

LW, AR, and YuL conceptualized the study. LW supervised the implementation of the first cohort. TT conducted data analyses of the first cohort and the functional studies. TT and YuL drafted the manuscript. YuL conducted the second cohort and the mouse study. XC analyzed exome sequencing data of the second cohort and the WES result of mice. YC helped with the benchwork of the first cohort. LJ and ZL participated in the subject enrollment of the first cohort. XH performed the Sanger sequencing assay of the second cohort and performed the qPCR assays. YiL and BW performed phenotyping analysis on a mouse study. RF supervised the second cohort, recruited the participants and was responsible for the mouse study. LW, ZY, RF, and AR critically revised the manuscript. All authors contributed to the article and approved the submitted version.

## Conflict of Interest

RF and BW formerly consulted with the now dissolved TeratOmic Consulting LLC. RF also receives travel funds to attend editorial board meetings of the Journal of Reproductive and Developmental Medicine published out of the Red Hospital of Fudan University. The remaining authors declare that the research was conducted in the absence of any commercial or financial relationships that could be construed as a potential conflict of interest.

## Footnotes

1 https://gatk.broadinstitute.org/hc/en-us

2 https://www.ncbi.nlm.nih.gov/snp

3 http://gnomad.broadinstitute.org/

4 http://exac.broadinstitute.org/

5 http://sift.jcvi.org/

6 http://genetics.bwh.harvard.edu/pph/

7 http://www.ebi.ac.uk/Tools/msa/clustalo/

8 http://pfam.xfam.org/
