# Supplementary material for: Somatic and de novo Germline Variants of MEDs in Human Neural Tube Defects
Source: Front Cell Dev Biol. 2021 Mar 4;9:641831. doi: 10.3389/fcell.2021.641831 (PMC7969791; doi:10.3389/fcell.2021.641831)
Supplement: Supplementary file 1 [file Data_Sheet_1.docx]

# Supplementary data

**Table S1 The basic characteristic of subjects.**

| **Sample ID** | **Phenotype** | **Gestational week** | **Gender** |
| --- | --- | --- | --- |
| A01 | SB | 28 | female |
| A02 | AN | 17 | unclear |
| A03 | SB | 13 | male |
| A04 | AN&SB | 37 | male |
| A05 | CRAN&AN | 16 | male |
| A06 | CRAN&AN | 28 | male |
| A07 | SB | 30 | male |
| A08 | SB | 27 | male |
| A09 | CRAN&AN | 24 | male |
| A10 | CRAN&AN | 16 | female |
| A11 | AN | 24 | female |
| A12 | AN | 17 | female |
| A13 | AN | 17 | female |
| A14 | AN | 28 | female |
| A15 | SB | 30 | female |
| A16 | CRAN&AN | 18 | female |
| A17 | CRAN&AN | 25 | female |
| A18 | AN&SB | 37 | female |
| A19 | CRAN&AN | 18 | female |
| A20 | AN&SB | 20 | female |
| A21 | AN | 21 | unclear |
| A22 | AN | 19 | male |
| A23 | SB | 38 | female |
| A24 | SB | 32 | female |
| A25 | SB | 28 | male |
| A26 | SB | 28 | male |
| A27 | AN | 33 | male |
| A28 | SB | 26 | female |
| B01 | CRAN | 23 | female |
| B02 | CRAN | 20 | unclear |
| B03 | SB | 16 | male |
| B04 | SB&AN | 33 | male |
| B05 | SB | 30 | female |
| B06 | SB | 22 | unclear |
| B07 | SB&AN | 20 | male |
| B08 | SB | 26 | female |
| B09 | SB | 28 | male |
| B10 | SB | 23 | female |
| B11 | SB | 21 | female |
| B12 | SB | 24 | female |
| B13 | SB&AN | 26 | male |
| B14 | SB | 25 | male |
| B15 | SB | 26 | male |
| B16 | CRAN | 17 | female |
| B17 | SB&AN | 21 | male |
| B18 | SB | 17 | male |
| B19 | SB | 23 | male |
| B20 | CRAN | 40 | male |

Note: SB: spinal bifida; CRAN: craniorachishisis ; AN: anencephaly

## Figure S1. Whole-exome sequencing on the CRISPR/Cas9 mice

WES sequencing was performed on three mouse embryos. Two of them were *Med12* ^p.Arg1782Cys^ mice (m1e1 and m1e2) and one was wildtype (m5e2). 23, 27, and 26 loss of function variants were detected in the three embryos, respectively. None of those genes overlapped with the 268 reported NTDs related genes. (A) Overlap map of detected variants with NTDs related genes. (B) The list of previously reported NTD-related genes.


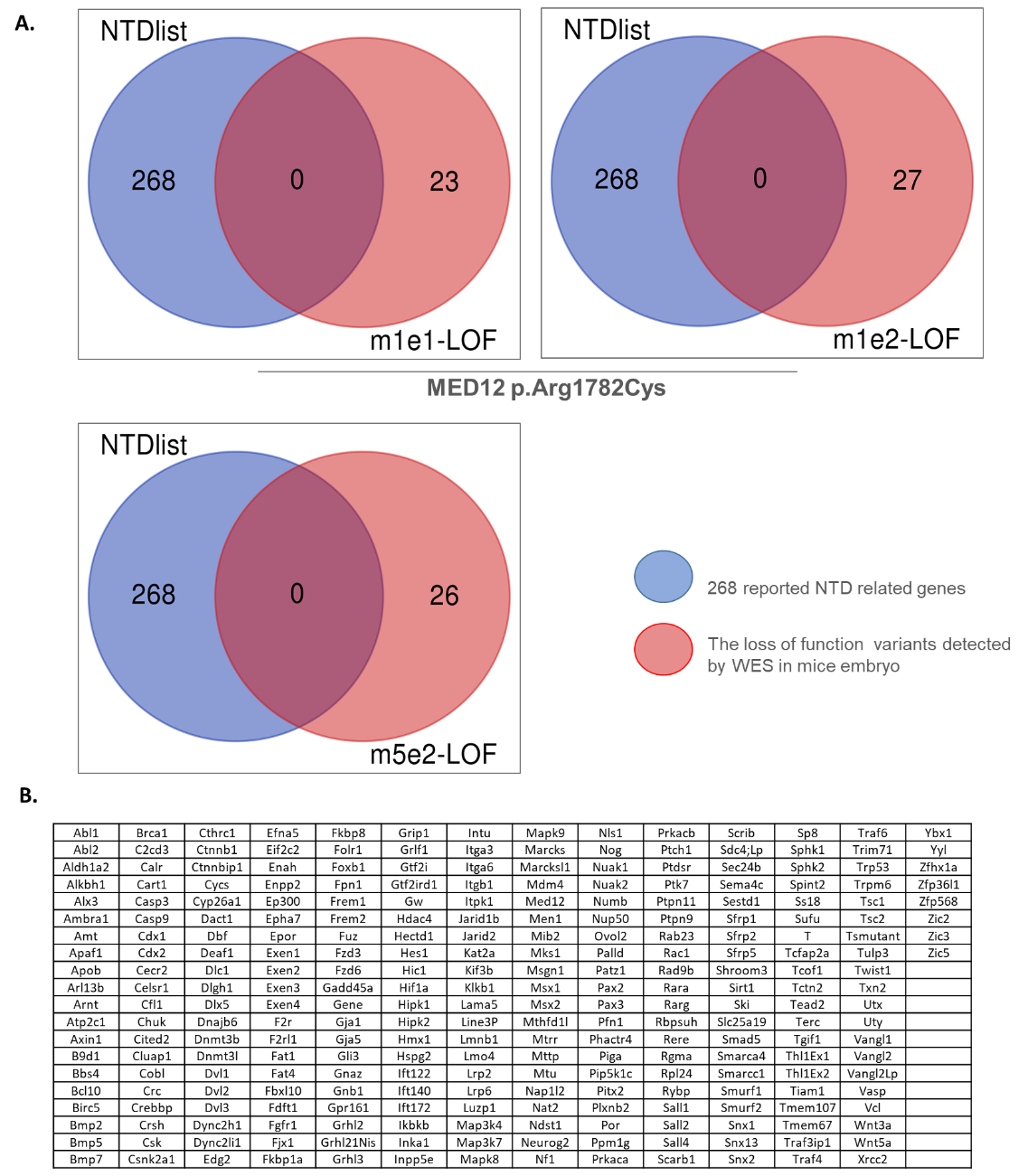


## Figure S2. Pathological examination

Pathological examinations (HE staining, 100x) of the spinal cord tissue from lesion site of spina bifida, the spinal cord tissue of control, and the brain tissue from anencephalic cases and control were performed. In the blue frame, the cells showed clear outline, but the nuclei were missing, indicating pyknosis, nuclear fragmentation, and karyolysis. The brain tissue of NTD cases also showed degradation. The cells of the neural system tissues from controls without an NTD phenotype were normal.


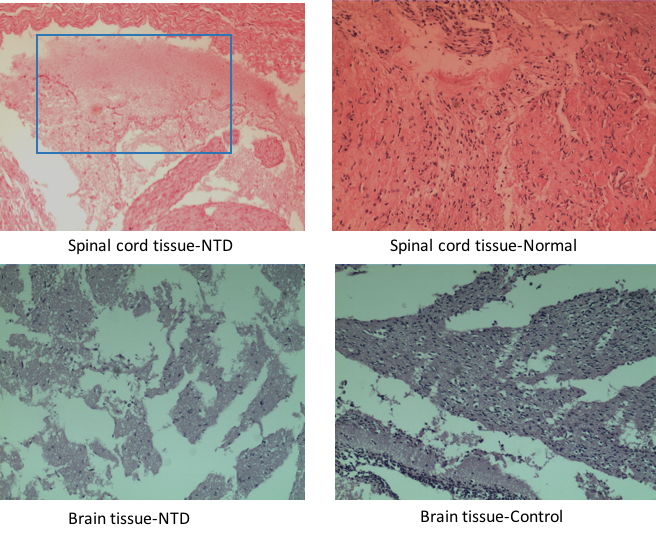


200um

## Figure S3. The relative expression of transfected plasmids by RT-qPCR assay

The MDCK-II cells were transfected with the same amount of Ampicillin resistant GFP-MED12 plasmids. We examined the expression of GFP and Ampicillin, the GAPDH and β-actin were used as the internal reference. The relative expression of GFP and Ampicillin to GAPDH and β-actin were calculated. Student-t test was used to compare the relative expression of plasmid RNA. As a result, none of the GFP/GAPDH or AMP/β-actin showed any significant difference between groups, suggesting relative expression of each WT and Mutant MED12 plasmids in cells was generally the same. Ns: no significant.
